# Patterns and Clinical Outcomes of Congenital Anomalies of the Kidney and Urinary Tract in Preterm Infants

**DOI:** 10.7759/cureus.88894

**Published:** 2025-07-28

**Authors:** Eman M AlFaraj, Hussain A Al Ghadeer, Zahra H Al-Sannaa, Mohammad H AlDar, Fatimah S Albattat

**Affiliations:** 1 Paediatrics, Qatif Central Hospital, Qatif , SAU; 2 Paediatrics, Maternity and Children Hospital, Al Ahsa, SAU; 3 Paediatrics, Qatif Central Hospital, Qatif, SAU; 4 Paediatrics, Maternity and Children Hospital, Dammam, SAU

**Keywords:** children, congenital anomalies, congenital anomalies of the kidney and urinary tract (cakut), kidney, saudi arabia, urinary tract

## Abstract

Introduction

Congenital anomalies of the kidneys and urinary tract (CAKUT) are embryonic disorders that arise during development and result in a spectrum of defects in the kidneys and outflow tracts, which include the ureters, the bladder, and the urethra. The clinical spectrum ranges from severe malformations, such as renal agenesis, to potentially milder manifestations, such as vesicoureteral reflux. CAKUT are the leading cause of kidney failure in children and one of the most common indications for kidney transplantation.

Purpose

This research aims to determine the pattern of CAKUT in preterm infants in Qatif Central Hospital, located in Qatif, which is in the eastern region of Saudi Arabia.

Methods

This cross-sectional study was performed to determine the pattern of CAKUT using ultrasound in neonates admitted to the neonatal intensive care unit (NICU) in Qatif Central Hospital in the Eastern region of Saudi Arabia between January 2018 and December 2022. Eligible participants were infants born at 23 to less than 37 weeks’ gestation. All neonates aged between one and 28 days of either gender admitted to the NICU with a postnatal diagnosis of congenital renal anomalies on an anomaly scan were included in the study.

Results

The presence of anomalies of the kidneys, ureters, bladder, or urethra was assessed and analyzed in a cross-sectional single-center study. Among the 48 patients, 30 (62.2%) were male and 18 (37.5%) were female, with a mean (SD) gestational age of 32.5 ± 3.5 weeks, and the majority of them had low birth weight (29, 60.4%). The most common findings related to CAKUT were hydronephrosis. The presence of CAKUT correlated with male gender, congenital heart disease, lung anomaly, and genetic disorders (p = <0.05).

Conclusion

The findings of this study suggest that clinicians caring for preterm infants should have a higher suspicion for CAKUT and consider screening, particularly those with extrarenal anomalies or genetic disorders, as preterm infants with CAKUT appear to be at significantly higher risk of death or severe illness. Detection of CAKUT can inform risk stratification and clinical decision-making and should also prompt clinicians to consider a genetic evaluation.

## Introduction

The prevalence of preterm birth is rising due to multiple factors such as advanced maternal age, assisted reproductive technology, and a concomitant increase in multiple gestations. The survival of preterm infants has substantially improved over the past decades [1]. When preterm infants are born, they are at risk of organogenesis disruption during their crucial time of development. Factors that play a role include inflammation, hypoperfusion, nephrotoxic medication, and antiangiogenic factors, which may result in structural and functional changes. The new advanced medical care and ongoing research have significantly improved the survival of preterm infants [2]. At the time of preterm delivery, most of the vital organs are still undergoing rapid development. Nephrogenesis is completed by 34 to 36 weeks of gestation [3]. Thus, it is likely that some alterations in the structural development of the kidneys could take place in preterm infants and impact their renal health, but clinical study data are limited. Further studies are required to determine the factors predisposing to renal malformation and to acknowledge the associated interventional strategies to optimize therapy and long-term renal health [4]. Impairment of fetal renal function can lead to decreased amniotic fluid, thus affecting fetal lung maturity by causing lung hypoplasia and early neonatal complications. In comparison to term infants, the preterm groups have a significantly reduced number of glomerular generations [5].

Risk factors influence the development of preterm kidney, including prenatal and postnatal events that may adversely affect the developing kidney, such as intrauterine and extrauterine growth restriction, antenatal medications, postnatal nephrotoxic medications, and hyperoxia. The benefit of antenatal glucocorticoids is by enhancing renal blood flow and glomerular filtration rate (GFR). Thus, nephrogenesis and renal maturation are affected [6]. The revolution and availability of high-resolution ultrasound equipment are increasingly contributing to the incidence of identifying congenital anomalies prenatally [7]. Congenital anomalies of the kidneys and urinary tract (CAKUT) is a diagnostic term for heterogeneous abnormalities, including changes in the kidney size, location, and parenchyma. In addition to the anomalies of the collecting system, the ureters, bladder, and urethra [8]. CAKUT is seen during routine antenatal ultrasound. Those findings may also be detected incidentally after the delivery. Etiologies are multifactorial, such as familial, genetic, epigenetic, exposure to teratogens, and environmental factors [9].

It’s a complex relationship between CAKUT and genetic disorders; individuals with the same genetic abnormalities can manifest different forms of CAKUT, and patients with the same phenotypic abnormalities might have distinct genetic disorders. Examples of genetic disorders associated with CAKUT include congenital heart disease, endocrinopathy, and immunodeficiencies [10, 11]. The presence of CAKUT should raise suspicion of an underlying genetic disorder. As well, CAKUT is considered to be a risk factor for morbidity and mortality. The standard care of preterm infants is to consider the risk-benefit in the management plan.

A study finding suggests a higher probability for serious illnesses frequently encountered in preterm infants with CAKUT, like sepsis, shock, acute kidney injury, necrotizing enterocolitis, and other serious illnesses. Longitudinal surveillance and prospective screening of preterm infants would clarify the risk of severe illness or mortality imparted by renal pathologies. Identifying specific gene-disease associations is important to improve clinical data and management [8]. The aim of the study is to identify the prevalence of CAKUT in preterm infants born in Qatif Central Hospital, located in Qatif, which is in the eastern region of Saudi Arabia. The associated congenital abnormalities and their impact on the clinical course of the patient.

## Materials and methods

Aim of the study

This study aims to determine the pattern and clinical outcomes of CAKUT in preterm infants admitted to the neonatal intensive care unit (NICU) at Qatif Central Hospital. Additionally, the study seeks to identify the associated underlying factors and outcomes of CAKUT in this population.

Study design and population

This research utilized an institution-based, retrospective, cross-sectional study design over five years, from 2018 to 2022. The study was conducted at Qatif Central Hospital, located in the eastern region of Saudi Arabia. The study population consisted of preterm neonates admitted to the NICU who were born between 23 and 36.6 weeks of gestational age. Only neonates with a birth weight greater than 450 grams were included in the study.

The inclusion criteria for this study were neonates who were less than 28 days old at the time of data collection, with a gestational age between 23 and 36+6 weeks of gestation, and a birth weight greater than 450 grams. The participants had to be admitted to the NICU at Qatif Central Hospital. These criteria ensured that the study focused on preterm neonates with sufficient clinical data and appropriate follow-up within the hospital setting.

Neonates were excluded from the study if they had missing or incomplete medical records, which would compromise data accuracy. Neonates with major congenital anomalies unrelated to the kidney or urinary tract, such as cardiac, neurological, or gastrointestinal anomalies, were also excluded, as these could confound the outcomes related to CAKUT. Additionally, neonates with a gestational age outside the specified range (i.e., less than 23 weeks or more than 36.6 weeks) or those with incomplete documentation regarding birth weight, gestational age, or CAKUT diagnosis were excluded. Neonates who were transferred to other hospitals before the completion of their NICU care were also excluded due to insufficient follow-up data.

Data collection

Data were collected retrospectively by reviewing the medical records of neonates born at Qatif Central Hospital and admitted to the NICU during the study period. Patient records were identified using the names and medical record numbers of the neonates. A structured data collection form was designed to capture essential demographic and clinical information, including the gestational age, birth weight, and any diagnosed congenital anomalies of the kidney and urinary tract. Data were systematically entered into a Microsoft Excel spreadsheet (Microsoft Corp., Redmond, WA), and all collected data were stored on a password-protected, encrypted universal serial bus (USB) memory stick to ensure confidentiality and security.

The data collected included the following: demographic information such as age, gender, and birth weight; clinical information, namely, gestational age, presence of CAKUT, and associated risk factors (e.g., maternal health, prenatal care); and outcome data such as short-term and long-term outcomes, including the need for surgical intervention, renal function outcomes, and any complications related to CAKUT.

Ethical considerations

The study was conducted in accordance with the ethical principles outlined in the Declaration of Helsinki. Ethical approval was obtained from the institutional review board (IRB) of Qatif Central Hospital prior to the initiation of the study (approval number: H-05-QT-115). Informed consent was not required due to the retrospective nature of the study, as all data were collected from anonymized patient records.

Data analysis

The data were collected, reviewed, and then fed to IBM SPSS Statistics software, version 26 (IBM Corp., Armonk, NY). All statistical methods used were two-tailed with an alpha level of 0.05, considering significance if the p-value was less than or equal to 0.05. Descriptive analysis for categorical data was done using frequencies and percentages, whereas numerical data were presented as mean with standard deviation. Also, the prevalence of CAKUT in detail and in total was graphed. Infants with associated renal anomalies and others besides antenatal risk factors were tabulated. Cross tabulation was done to show factors associated with CAKUT and to test the association between CAKUT and clinical outcomes using the Pearson chi-square test and exact probability test for small frequency distributions.

## Results

A total of 48 eligible preterm infants were reviewed and assessed, with their gestational age ranging from 23 to 36+6 weeks, with a mean gestational age of 32.5 ± 3.5 weeks. Considering birth weight, four (8.3%) infants had an extremely low birth weight, 12 (25%) had very low birth weight, 29 (60.4%) had low birth weight, and three (6.3%) had normal birth weight. Thirty-eight (79.2%) infants needed mechanical ventilation (Table 1).

**Table 1:** Biodemographic data of the preterm infants in the study group (n=48)

Biodemographic data	Number	Percentage (%)
Gender		
Male	30	62.5%
Female	18	37.5%
Gestational age (by weeks)		
< 35 weeks	28	58.3%
> 35 weeks	20	41.7%
Mean ± SD	32.5 ± 3.5	
Birth weight		
Extremely low<1kg	4	8.3%
Very low <1.5	12	25.0%
Low <2.5	29	60.4%
Normal	3	6.3%
Need for mechanical ventilation		
Yes	38	79.2%
No	10	20.8%

**Figure 1:**
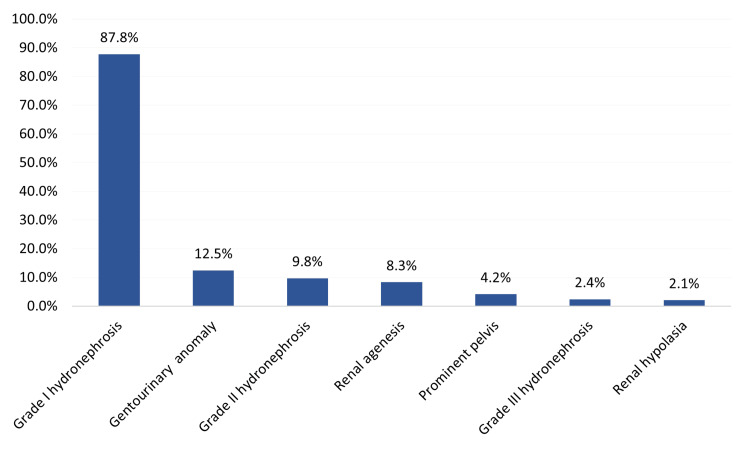
Congenital anomalies of the kidney and urinary tract (CAKUT) in preterm infants in the study group

A total of 10 (20.8%) preterm infants had at least one type of CAKUT. In more detail, 36 (87.8%) had grade I hydronephrosis, six (12.5%) had genitourinary anomalies, four (9.8%) had grade II hydronephrosis, four (8.3%) had renal agenesis, two (4.2%) had prominent pelvis, and one infant (2.1%) had renal hypoplasia (Figure 1).

**Figure 2:**
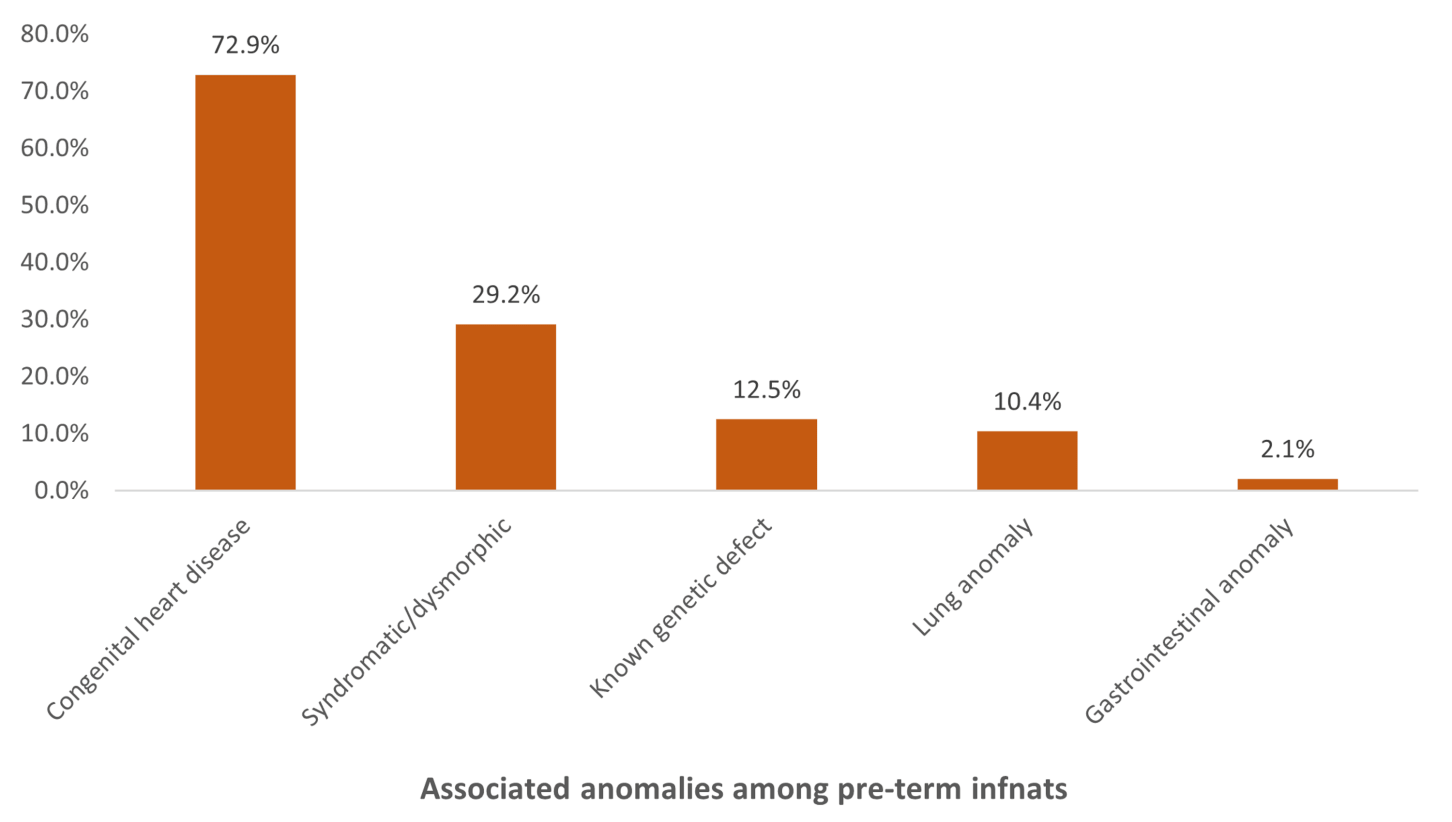
Associated anomalies found in the preterm infants in the study group

As for associated anomalies (Figure 2), the most reported anomalies were congenital heart disease (35, 72.9%), syndromic dysmorphic anomalies (14, 29.2%), known genetic defects (six, 12.5%), lung anomalies (five, 10.4%), and gastrointestinal anomalies (one, 2.1%).

Table 2 shows the laboratory data among the preterm infants included in the study. Only three (6.3%) infants had high creatinine levels, and one (2.1%) had a urinary tract infection that was associated with CAKUT.

**Table 2:** Laboratory data of the preterm infants in the study group CAKUT: congenital anomalies of the kidneys and urinary tract

Laboratory parameters	Number	Percentage (%)
High creatinine		
Yes	3	6.3%
No	45	93.8%
Urinary tract infection associated with CAKUT		
Yes	1	2.1%
No	47	97.9%

Table 3 reveals the distribution of high creatinine levels and urinary tract infections among the study group by their reported CAKUT. All cases with renal hypoplasia had high creatinine levels, as did two (33.3%) of the others with genitourinary anomaly and two (5.6%) of cases with grade I hydronephrosis. Urinary tract infections were reported in only one case with grade I hydronephrosis.

**Table 3:** Distribution of high creatinine level and urinary tract infection among the study sample by their reported CAKUT CAKUT: congenital anomalies of the kidneys and urinary tract

CAKUT	High creatinine		Urinary tract infection associated with CAKUT	
	Number	Percentage (%)	Number	Percentage (%)
Genitourinary anomaly				
Yes	2	33.3%	0	0.0%
No	1	2.4%	1	2.4%
Renal agenesis				
Yes	0	0.0%	0	0.0%
No	3	6.8%	1	2.3%
Renal hypoplasia				
Yes	1	100.0%	0	0.0%
No	2	4.3%	1	2.1%
Prominent pelvis				
Yes	0	0.0%	0	0.0%
No	3	6.5%	1	2.2%
Hydronephrosis grade				
Grade I	2	5.6%	1	2.8%
Grade II	0	0.0%	0	0.0%
Grade III	0	0.0%	0	0.0%

Table 4 presents the antenatal risk factors among the study sample. The most reported antenatal risk factors included maternal disease (15, 31.3%), amniotic fluid abnormalities (11, 22.9%), diabetes mellitus (nine, 18.8%), sickle cell disease (six, 12.5%), and renal disorder (one, 2.1%).

**Table 4:** Antenatal risk factors among the preterm infants in the study group

Risk factors	Yes		No	
	Number	Percentage (%)	Number	Percentage (%)
Amniotic fluid abnormalities	11	22.9%	37	77.1%
Chorioamnionitis	0	0.0%	48	100.0%
Maternal disease	15	31.3%	33	68.8%
Antiphospholipid	0	0.0%	48	100.0%
Thyroid	3	6.3%	45	93.8%
Cardiac	0	0.0%	48	100.0%
Sickle cell disease (SCD)	6	12.5%	42	87.5%
Renal	1	2.1%	47	97.9%
Diabetes	9	18.8%	39	81.3%
Smoker	0	0.0%	48	100.0%

Table 5 presents the clinical outcomes of the preterm infants in the study group. As for hospital stay, 15 (31.3%) stayed for seven to 14 days, while 14 (29.2%) stayed for more than 30 days. The in-hospital mortality rate was nine (18.8%), while 31 (64.6%) improved.

**Table 5:** Clinical outcomes of the preterm infants in the study group

Outcome	Number	Percentage (%)
Length of stay (by days)		
7-14	15	31.3%
15-30	19	39.6%
> 30	14	29.2%
Mean ± SD	26.3 ± 20.8	
Clinical outcome		
Improved	31	64.6%
Transferred to other hospitals	7	14.6%
Discharge against medical advice	1	2.1%
In-hospital death	9	18.8%

Table 6 shows the relationship between CAKUT in preterm infants and hospital stay in days. None of the CAKUT were significantly associated with prolonged hospital stay. Only 12 (33.3%) of cases with grade I hydronephrosis stayed for more than 30 days versus one (25%) of others with grade II (P=.755).

**Table 6:** Relationship between CAKUT in the study group and hospital stay (in days) P: exact probability test; CAKUT: congenital anomalies of the kidney and urinary tract

CAKUT	Length of hospital stay (by days)						p-value
	7-14		15-30		> 30		
	Number	Percentage (%)	Number	Percentage (%)	Number	Percentage (%)	
Genitourinary anomaly							.228
Yes	3	50.0%	3	50.0%	0	0.0%	
No	12	28.6%	16	38.1%	14	33.3%	
Renal agenesis							.390
Yes	2	50.0%	2	50.0%	0	0.0%	
No	13	29.5%	17	38.6%	14	31.8%	
Renal hypoplasia							.325
Yes	1	100.0%	0	0.0%	0	0.0%	
No	14	29.8%	19	40.4%	14	29.8%	
Prominent pelvis							.203
Yes	0	0.0%	2	100.0%	0	0.0%	
No	15	32.6%	17	37.0%	14	30.4%	
Hydronephrosis grade							.755
Grade I	11	30.6%	13	36.1%	12	33.3%	
Grade II	1	25.0%	2	50.0%	1	25.0%	
Grade III	0	0.0%	1	100.0%	0	0.0%	

Table 7 showcases the relationship between the CAKUT in the study group and clinical outcomes. Three (75%) of cases with renal agenesis died compared to six (13.6%) others without (P=.010). Furthermore, one case with renal hypoplasia died (P=.043).

**Table 7:** Relation between CAKUT in the study group and clinical outcomes P: exact probability test; CAKUT: congenital anomalies of the kidneys and urinary tract; *P < 0.05 (significant)

CAKUT	Clinical outcomes						p-value
	Improved		Transferred		Died		
	Number	Percentage (%)	Number	Percentage (%)	Number	Percentage (%)	
Genitourinary anomaly							.382
Yes	4	66.7%	0	0.0%	2	33.3%	
No	27	64.3%	8	19.0%	7	16.7%	
Renal agenesis							.010*
Yes	1	25.0%	0	0.0%	3	75.0%	
No	30	68.2%	8	18.2%	6	13.6%	
Renal hypoplasia							.043*
Yes	0	0.0%	0	0.0%	1	100.0%	
No	31	66.0%	8	17.0%	8	17.0%	
Prominent pelvis							.472
Yes	1	50.0%	0	0.0%	1	50.0%	
No	30	65.2%	8	17.4%	8	17.4%	
Hydronephrosis grade							.159
Grade I	27	75.0%	6	16.7%	3	8.3%	
Grade II	3	75.0%	0	0.0%	1	25.0%	
Grade III	0	0.0%	1	100.0%	0	0.0%	

Table 8 shows the factors associated with CAKUT among preterm infants. A total of nine (30%) male infants had CAKUT versus one (5.6%) female (P=.044). Also, two (50%) infants with grade II hydronephrosis had CAKUT compared to none of grade III (P=.017). CAKUT was detected in two (66.7%) infants with high creatinine compared to eight (17.8%) with other conditions (P=.044). Ten (28.6%) infants with congenital heart disease had CAKUT, 60% of infants with lung anomalies, six (42.9%) of infants with syndromic/dysmorphic anomalies, and all cases with renal anomalies (P<0.05).

**Table 8:** Factors associated with CAKUT among preterm infants P: exact probability test; CAKUT: congenital anomalies of the kidneys and urinary tract; *P < 0.05 (significant)

Factors		Any CAKUT				p-value
		Yes		No		
		No	%	No	%	
Gender	Male	9	30.0%	21	70.0%	.044*
	Female	1	5.6%	17	94.4%	
Gestational age (by weeks)	< 35 weeks	6	21.4%	22	78.6%	.904
	> 35 weeks	4	20.0%	16	80.0%	
Birth weight	Extremely low<1kg	1	25.0%	3	75.0%	.647
	Very low <1.5	1	8.3%	11	91.7%	
	Low <2.5	7	24.1%	22	75.9%	
	Normal	1	33.3%	2	66.7%	
Need of mechanical ventilation	Yes	9	23.7%	29	76.3%	.343
	No	1	10.0%	9	90.0%	
Hydronephrosis grade	Grade I	2	5.6%	34	94.4%	.017*
	Grade II	2	50.0%	2	50.0%	
	Grade III	0	0.0%	1	100.0%	
High Creatinine	Yes	2	66.7%	1	33.3%	.044*
	No	8	17.8%	37	82.2%	
Urinary tract infection associated with CAKUT	Yes	0	0.0%	1	100.0%	.604
	No	10	21.3%	37	78.7%	
Congenital heart disease	Yes	10	28.6%	25	71.4%	.030*
	No	0	0.0%	13	100.0%	
Lung anomaly	Yes	3	60.0%	2	40.0%	.023*
	No	7	16.3%	36	83.7%	
Gastrointestinal anomaly	Yes	0	0.0%	1	100.0%	.604
	No	10	21.3%	37	78.7%	
Syndromatic/dysmorphic	Yes	6	42.9%	8	57.1%	.016*
	No	4	11.8%	30	88.2%	
Known genetic defect	Yes	2	33.3%	4	66.7%	.646
	No	8	19.5%	33	80.5%	
Amniotic fluid abnormalities	Yes	4	36.4%	7	63.6%	.149
	No	6	16.2%	31	83.8%	
Sickle cell disease (SCD)	Yes	0	0.0%	6	100.0%	.179
	No	10	23.8%	32	76.2%	
Renal	Yes	1	100.0%	0	0.0%	.049*
	No	9	19.1%	38	80.9%	
Diabetes	Yes	2	22.2%	7	77.8%	.909
	No	8	20.5%	31	79.5%	

## Discussion

The current study aimed to identify the pattern of CAKUT in preterm infants born in Qatif Central Hospital and study the associated congenital abnormalities and their impact on the clinical course of the patient. Kidney failure in children is mostly caused by CAKUT, which is also one of the most common reasons for kidney transplantation [12]. The spectrum of morphologic phenotypes is broad, encompassing milder disorders like dilatation of the urinary tract to more severe deformities like bilateral kidney agenesis. According to estimates, there are four to 60 cases of CAKUT for every 10,000 live births [13]. When an infant exhibits related clinical signs and symptoms, CAKUT is frequently identified either postnatally or during normal prenatal ultrasound imaging. Diagnoses by accident could also happen. Multifactorial etiologies comprise environmental, genetic, family, and epigenetic influences [14].

The current study revealed that most preterm infants were male, with gestational age less than 35 weeks and low birth weight. Also, more than three-fourths needed mechanical ventilation. Considering the prevalence of CAKUT, one-fifth of the preterm infants had at least one type of kidney and urinary tract anomaly, mainly genitourinary anomalies, renal agenesis, prominent pelvis, and renal hypoplasia. Also, about three-fourths had an associated congenital heart disease. Grade I hydronephrosis was the most detected grade, but very few infants had high creatinine levels or urinary tract infections associated with CAKUT. A much lower prevalence was reported by Mohamed et al. [15], where the prevalence of CAKUT was 2% in preterm infants. Another study by Hays et al. [16] found that 2% of the preterm infants had CAKUT, with urinary tract dilation comprising the majority of cases (70%). Leow et al. [17] documented that the prevalence of CAKUT was 1.5% among infants hospitalized in 419 NICUs. Among the 13,383 infants with CAKUT analyzed, median gestational age was 35 (interquartile range (IQR) 31-38) weeks, and median birth weight was 2.34 (IQR 1.54-3.08) kg. A slightly higher prevalence was reported by Janchevska et al. [18], as 7% of small for gestational age (SGA) born children had congenital anomalies of the urinary tract. Their mean birth weight was very low (1855 g, -3.93 SDS). Li et al. [19] found that the average prevalence of CAKUT in preterm infants born to mothers overall and mothers aged ≥35 years was around 1.60 per 1,000 births. They also found that the prevalence of CAKUT changed over time among all women and women of advanced maternal age, although no significant trends were observed. CAKUT were more likely to occur in male than female newborns, which is consistent with the current study's findings. The prevalence of CAKUT ranges from 4.2 per 10,000 births in Taiwan to 4.0 per 1,000 births in Russia and some Asian and European countries [20-24]. The increasing prevalence might be owing to increased screening, developments in ultrasound technology, and improved birth defect surveillance in our study. The upward trend in prevalence of CAKUT in Europe agrees with this view [25,26].

The current study also revealed that male gender, having other congenital anomalies, and maternal renal disorders were associated with developing CAKUT among their preterm infants. Similar to the current study findings, other studies revealed that CAKUT are likely to be associated with malfunctions in other organs [24, 27]. Other risk factors were assessed by the literature, including oligohydramnios, extra-renal anomalies, peak serum creatinine, and exposure to nephrotoxic medications [17]. In contrast to the literature, gestational age and birth weight were insignificantly associated with CAKUT [28-31].

Regarding the clinical outcome, the current study revealed a high hospital admission duration among preterm infants with CAKUT, mostly for two weeks to one month. Our study has a lower mortality rate compared to Leow et al.'s study [6]. 

Limitation

Due to its retrospective nature, our study has several limitations. We lacked information on prenatal ultrasound evaluations. Furthermore, there were far too few patients. Not every patient in the present research underwent micturating cystourethrogram (MCUG). Our study's failure to follow up with various CAKUT subgroups is another limitation.

## Conclusions

The current study revealed that nearly one out of every five preterm infants had at least one type of CAKUT, with a higher rate among male children with other congenital anomalies and maternal-associated renal disorders. It is important to regularly check for congenital anomalies, especially those involving the cardiovascular system, by performing CAKUT screenings. Following the diagnosis of CAKUTs, recommendations for postnatal care and a thorough evaluation of any coexisting defects should be given.

This study provides valuable insights into the prevalence of CAKUT in preterm infants, specifically within Saudi Arabia. The use of ultrasound as a diagnostic tool ensures reliable detection of CAKUT, and the inclusion of a wide range of gestational ages (23 to less than 37 weeks) captures a broad spectrum of CAKUT manifestations. The study also highlights important risk factors, including gender, congenital heart disease, lung anomalies, and genetic disorders, which help identify high-risk populations. With a substantial sample size of 48 patients, the study offers robust data that can inform clinical practice and future research on CAKUT in preterm infants.
